# Investigating Glutamatergic Mechanism in Attention and Impulse Control Using Rats in a Modified 5-Choice Serial Reaction Time Task

**DOI:** 10.1371/journal.pone.0115374

**Published:** 2014-12-19

**Authors:** Abigail Benn, Emma S. J. Robinson

**Affiliations:** Department of Physiology and Pharmacology, School of Medical and Veterinary Sciences, University of Bristol, Bristol, United Kingdom; Queen Mary University of London, United Kingdom

## Abstract

The 5-choice serial reaction time task (5CSRTT) has been widely used to study attention and impulse control in rodents. In order to mimic cognitive impairments in psychiatry, one approach has been to use acute administration of NMDA antagonists. This disruption in glutamatergic transmission leads to impairments in accuracy, omissions, and premature responses although findings have been inconsistent. In this study, we further investigated glutamatergic mechanisms using a novel version of the 5CSRTT, which we have previously shown to be more sensitive to cognitive enhancers. We first investigated the effects of systemic treatment with NMDA antagonists. We also carried out a preliminary investigation using targeted medial prefrontal cortex infusions of a NMDA antagonist (MK801), mGluR2/3 antagonist (LY341495), and mGluR7 negative allosteric modulator (MMPIP). Acute systemic administration of the different NMDA antagonists had no specific effects on accuracy. At higher doses PCP, ketamine, and memantine, increased omissions and affected other measures suggesting a general disruption in task performance. Only MK801 increased premature responses, and reduced omissions at lower doses suggesting stimulant like effects. None of the NMDA antagonists affected accuracy or any other measures when tested using a short stimulus challenge. Infusions of MK801 had no effect on accuracy but increased premature responses following infralimbic, but not prelimbic infusion. LY341495 had no effects in either brain region but a decrease in accuracy was observed following prelimbic infusion of MMPIP. Contrary to our hypothesis, disruptions to glutamate transmission using NMDA antagonists did not induce any clear deficits in accuracy in this modified version of the 5CSRTT. We also found that the profile of effects for MK801 differed from those observed with PCP, ketamine, and memantine. The effects of MK801 in the infralimbic cortex add to the literature indicating this brain region and glutamate play an important role in impulse control.

## Introduction

Studies in both healthy human volunteers and normal animals have found that acute treatments with NMDA antagonists induce a range of behavioural and cognitive impairments [1]–[5]. The profile of impairments induced by NMDA antagonism in healthy human volunteers is similar to those seen in Schizophrenia [1], [3], [5]. There is also evidence that NMDA antagonism exacerbates cognitive deficits in schizophrenic patients [6]. These observations underpin the use of both acute and chronic treatment with different NMDA antagonists as an approach to model cognitive impairments in psychiatry in animals [2], [7]–[11]. These studies include animal studies investigating deficits in visuo-spatial attention in the 5-choice serial reaction time task (5CSRTT), which is derived from the human continuous performance task [12]. However, inconsistencies in the presentation of specific cognitive impairments have raised concerns about the predictive validity of using NMDA antagonism to model cognitive impairments in animals [4], [8], [10]. This was particularly evident in a study by Smith et al where direct comparison of different NMDA antagonists found both compound and task-dependent differences [4]. This study also reported that effects were generally confounded by non-specific effects on motor and/or motivational behaviours.

The 5CSRTT is used to study visuo-spatial attention and impulse control in rodents [12]. Systemic and locally targeted NMDA antagonists typically induce impairments in accuracy, omissions, and impulsive responding in the 5CSRTT although there is a lack of consistency in the results from within and across laboratories [4], [13], [14]. Following systemic treatment, most studies observe impairments in attention concomitant with more general disruption to task performance [4], [7], [15]–[17]. Using targeted brain microinfusions, a more specific profile of impairments have been observed [13], [14]. Infusions into the prelimbic cortex are linked to impairments in attention whilst infusions into the infralimbic cortex have been linked to impulsivity [13]. These performance deficits have also been related to elevations in cortical glutamate release, thought to involve GABA interneurone mediated disinhibition of pyramidal neurones [18]–[21].

The aim of this study was to investigate the effects of glutamate receptor manipulations using a variable inter-trial interval (VITI) version of the 5CSRTT, where stimulus presentation is delivered in an un-predictable manner throughout training and testing. We, along with other groups, have recently developed novel versions of the 5CSRTT designed to enhance the sensitivity of the method to attentional deficits and cognitive enhancers [22]–[25]. Extended training periods are required for optimal performance in behavioural tasks such as the 5CSRTT. In the original version of the task, a fixed inter-trial interval (ITI) is used (usually 5 s). This has been hypothesised to lead to the development of behavioural strategies in well-trained rats which enable them to predict the timing of the light cue [2], [26]. In this study, we have used a VITI task in which the rats are trained and tested in a task where the presentation of the light cue is not as readily predicted. We have previously shown that this task is sensitive to the attentional benefits induced by oral methylphenidate and atomoxetine, something which was not as readily observed in the standard version of the task [22]. In order to test whether this non predictable presentation of the light cue increased the attentional impairments induced by acute NMDA antagonists, we tested four different non-competitive NMDA antagonists (ketamine, PCP, MK801, and memantine) which have all previously been used in the standard 5CSRTT. Previous studies using NMDA/glutamate manipulations have used the VITI schedule as an acute manipulation in the standard 5CSRTT, designed to increase the demands of the task [17], [24], [25]. Interestingly, these studies have found that specific antagonism of NR2B NMDA receptors can improve 5CSRTT performance under such conditions [17], as opposed to MK801 that showed no effect [24]. In addition, competitive NMDA antagonism has shown no effect on visuo-spatial attention or impulse control under a VITI training schedule with single doses of r-CPP [27], despite the induction of performance deficits with repeated exposure to PCP [25].

In the second part of the study, we build on the work of Murphy et al, [13], [14] to investigate the effects of direct modulation of glutamate neurotransmission in the medial prefrontal cortex (mPFC). In this study, treatments were targeted to the prelimbic and infralimbic cortices which have previously been shown to modulate dissociable effects on attention (prelimbic) and impulse control (infralimbic). In these experiments, we used the NMDA antagonist, MK801, the orthosteric mGluR2/3 antagonist, LY341495, and mGluR7 negative allosteric modulator, MMPIP [28]. We included mGluR manipulations to investigate whether treatments which facilitate cortical glutamate release would induce similar deficits to those observed following NMDA antagonist infusions. In a previous study, infusions of a selective glutamate reuptake inhibitor (DL-TBOA), or lamotrigine, an anti-epileptic drug which attenuates excess glutamate release, failed to show any impairment [14]. LY341495 facilitates the release of glutamate [29] through receptors located pre- and post-synaptically [30], whilst MMPIP blocks pre-synaptic mGluR7 located on glutamate terminals within the active zone of neurotransmitter release [28].

## Materials and Methods

### Subjects

All studies used male, Lister Hooded rats weighing 300–350 g (Harlan, UK) at the start of the experiments and 400–450 g at the start of dosing. Separate cohorts of animals were used for the systemic drug administration (experiment 1 and 2, n = 12) and infusion experiments (experiment 3, n = 12). Animals were housed in pairs under temperature controlled conditions and 12-h: 12-h reverse light-dark cycle (lights off at 0700 hours). Behavioural testing took place during the animals' active phase, between 09:00 and 19:00 h, 5 days/week. Rats were maintained at approximately 90% of their free feeding weight (∼18 g/day per rat) on laboratory chow (Purina, UK), water was provided *ad libitum*. All experiments were conducted in accordance with the UK Animals (Scientific Procedures) Act 1986, and were approved by the local ethical review group (University of Bristol). All surgery was performed under isoflurane anaesthesia, euthanasia was performed using an overdose of sodium pentobarbitone; all efforts were made to minimize suffering.

### Drugs

All drugs were bought from Tocris, UK. For systemic administration drugs used were; ketamine hydrochloride, (+)-MK801 maleate, phencyclidine hydrochloride, and memantine hydrochloride. Drugs were prepared fresh every day, dissolved in 0.9% saline, and administered by intraperitoneal injection in a final volume of 1 ml/kg. Drugs were delivered 10 min (ketamine), 30 min (MK801), 40 min (PCP), and 60 min (memantine) before behavioural testing began in accordance with previous reports [4].

For mPFC infusion experiments, the following drugs were used targeted to the prelimbic and infralimbic cortices and delivered in a final volume of 1 µL per hemisphere; MMPIP hydrochloride [6-(4-Methoxyphenyl)-5-methyl-3-(4-pyridinyl)-isoxazolo(4,5-c)pyridin-4(5H)-one], LY341495 [(2S)-2-Amino-2-[(1S,2S)-2-carboxycycloprop-1-yl]-3-(xanth-9-yl) propanoic acid], and MK801. Drugs were dissolved in 25% 2-hydroxypropyl-β-cyclodextrin (MMPIP and LY341495) or PBS (MK801) and stored at −20°C.

### Behavioural procedures

Training and testing was conducted in rat five-hole operant boxes manufactured by Med Associates, USA (Sandown Scientific, UK), and controlled by KLimbic Software (Conclusive Solutions Ltd, UK). Rats were trained, in a modified version of the 5CSRTT [22], to initiate a trial by making a nose poke response in the magazine. After the VITI period of 5, 6, or 7 s, animals' responded to a brief light cue (0.5 s). The timings for the VITI were based on previous studies where this approach has been used as an acute manipulation in the standard 5CSRTT [31], [32]. Both the ITI and position of the light cue were presented in a pseudorandom order, generated by the operant software. A correct response was rewarded with a single reward pellet (45 mg Noyes Precision Pellet, Sandown Scientific, UK). Subjects performed the task in the dark, with the house light illuminated as a 5 s punishment following an incorrect response (response in the wrong aperture), omission (no response during the limited hold period of 5 s), or premature response (response before the presentation of the light cue). Animals were trained according to a 12 stage protocol [22], [31] with the stimulus duration gradually reduced from 30 to 0.5 s (5–7 sessions per week). Each session consisted of 105 completed trials and lasted a maximum of 30 min. Animals were trained until a stable performance was reached (>80% accuracy, <20% omissions). Once criterion was reached (after approx. 10 weeks) animals were run for a further 10 sessions before any manipulation was carried out.

### Surgery

Following training, animals (n = 12) were implanted with an intra-cerebral guide cannula to facilitate infusions into the mPFC. Both animals in the cage pair received the same surgical manipulation but subsequent infusions were fully counter-balanced across the whole cohort. Surgery was performed under aseptic conditions. Animals were anaesthetized with inhaled isoflurane in medical oxygen, (induction 5%, maintenance 2%, flow rate 2 L/min) and placed in a stereotaxic frame (David Kopf Instruments, USA) fitted with a nose cone for continuous delivery of anaesthetic. The skull was exposed and intraepicaine (2%, Dechra Ltd, Staffordshire, UK) was administered locally for post-operative analgesia. Two small burr holes were drilled through the skull, and bilateral 22-gauge stainless steel cannula, 1.5 mm separation (Plastics One, Sevenoaks, UK), implanted into the mPFC, and secured to the skull with stainless steel bone screws and dental acrylic. The following stereotaxic coordinates were used relative to bregma, anteroposterior +3.0 mm, lateromedial ±0.75 mm, and dorsoventral −2.2 mm (Paxinos and Watson 2007). Internal obturators were inserted flush to the bottom of the guide cannula. An aluminium cap was then fitted to further protect the cannula system. Following surgery animals were housed in pairs and given 5–7 days recovery with free access to food and water.

### Microinfusion procedure

Following post-operative re-baseline sessions (10 sessions) animals were habituated to the infusion procedure. Subjects were minimally restrained and the obturators removed and cleaned with 70% isopropyl alcohol pre-injection swabs (Robinson Healthcare, UK), before being replaced with 28-gauge bilateral infusion cannula that extended into the prelimbic cortex (1.5 mm projection from the end of the guide). The injector was also cleaned with isopropyl alcohol and allowed to dry prior to use. The animals received two habituation sessions where the injector was inserted but no infusion was carried out before the experiment started. Drugs were infused into the prelimbic cortex, as detailed below, with the animal receiving at least four days with no manipulations between each of the drug treatments used. Animals were then given two weeks off and a two week re-baseline before commencing the infralimbic infusions, where the habituation procedure was repeated. Infralimbic internal cannula extended 3 mm beyond the end of the guide cannula. During the infusion procedure, the bilateral injector was left in place for 1 min before drug or vehicle delivery. Drugs were delivered in a total volume of 1 µL over 2 min. Based on published methodology targeting the same brain regions [13], this volume was expected to be limited to the appropriate target regions with the estimated spread in the range of 1–1.5 mm^3^ [33]. The injector was left for a further 2 min after the infusion to allow diffusion of the liquid into the surrounding tissue. The injector was then removed and replaced with the obturator and the metal skull cap. The animal was then left for 5 min in the company of other animals before being placed in the operant chamber and the behavioural protocol started.

### Experiment 1: Effects of systemic treatment with NMDA antagonists on performance in a VITI 5CSRTT

Using a within-subject study design (n = 12 animals) dose-response experiments were performed for ketamine (0.0, 0.1, 0.3, 1.0 mg/kg, i.p.), MK801 (0.0, 0.01, 0.03, 0.1 mg/kg, i.p.), PCP (0.0, 0.3, 1.0, 3.0 mg/kg, i.p.), and memantine (0.0, 0.1, 1.0, 3.0 mg/kg, i.p.). Higher doses for ketamine were also tested (0.0, 3.0, 6.0 mg/kg, i.p.) after the initial dose response study revealed no main effects on behaviour. Animals received drug or vehicle in a fully counterbalanced Latin square design. Doses were administered twice a week (Tuesday and Friday), with baseline sessions run on the days preceding dosing days (Monday and Thursday). A washout period of at least 4 days was used after each drug.

### Experiment 2: Effects of systemic treatment with NMDA antagonists during a short stimulus challenge in the VITI 5CSRTT

Following completion of the initial dose-response experiments and following 1 week without treatments, a short-stimulus challenge (SSC) experiment was carried out. Animals were run under the VITI protocol (0.5 s stimulus duration) for 4 consecutive days before the stimulus duration was reduced to 0.25 s on the 5^th^ day for testing. This schedule was repeated for each compound tested versus vehicle control, counter-balanced. The SSC session was used once per week to reduce adaptations in the animals' performance. Single doses of ketamine (1.0 mg/kg, i.p.), MK801 (0.03 mg/kg, i.p.), and PCP (0.3 mg/kg, i.p.) were chosen based on the highest dose which did not induce general impairments in the previous dose response experiment.

### Experiment 3: Effects of glutamate receptor blockade in prelimbic versus infralimbic cortex in a VITI 5CSRTT

Each drug was tested against its own vehicle control in a series of dose-response experiments. Each experiment used a fully counter-balanced study design with the following experiments carried out: MMPIP (0.0, 0.1, 1.0 µg/µL), LY341495 (0.0, 0.3, 1.0 µg/µL), and MK801 (0.0, 3.0, 10.0 µg/µL). At the end of these experiments a higher dose of LY341495 was infused into the prelimbic cortex at a concentration of 10.0 µg/µL versus vehicle control after no effects were found in the initial dose response experiment. The higher dose was then also used for subsequent infralimbic infusions (0.0, 1.0, 10.0 µg/µL). Infusion studies were run in 2×3-day cycles per week, starting with a baseline session, then a drug or vehicle infusion 5 min before testing in the operant chambers. On the third day, animals were given a day off and remained in their home cage. Animals were removed from the study if cannula position was found to be incorrect, or if the extent of tissue damage suggested infection and was beyond the localised region of the injection site. Animals were also removed due to head mount failure; these animals did not complete the full study. Two animals had head mount failure before drug dosing commenced. A total of 4 animals were removed during the course of the experiment, 2 for head mount failure, and 2 due to incorrect placement. Animal numbers for the final analysis for each drug infusion were as follows; prelimbic, MMPIP n = 10, LY341495 and MK801 n = 9, infralimbic, n = 6 for all drugs.

### Histology

At the end of the experiment, cannulated animals were euthanized by anaesthetic overdose, the brains removed and post fixed in 4% paraformaldehyde. After fixation, the brains were left to sink in 30% sucrose solution made up in 0.1 M PBS, before 40 µm coronal sections were cut using a freezing microtome. Sections were then stained with cresyl violet and the injector tip position mapped onto standardized coronal sections of a rat brain stereotaxic atlas (Paxinos and Watson 2007).

### Statistical Analysis

The design of the experiments and analysis were based on our previous methodology and those of similar published studies and in line with the principles set out in Cardinal and Aitken, 2006. The dose-response experiments for each treatment (systemic or microinfusion) included their own control and were analysed as independent experiments with animals given a minimum of 4 days washout between drug treatments. For the infusion studies, there was a two week period between the prelimbic and infralimbic infusions during which time the animals were given 7 days off and 5 session's re-baseline. The following performance measures were recorded, accuracy (%, number of correct responses divided by the total number of correct and incorrect responses*100), the number of correct responses, omissions (%, number of omission responses, divided by the total number of correct, incorrect and omissions*100), premature responses (%, number of premature responses divided by the total number of correct, incorrect, and omissions*100), correct latency (time taken to make a correct response in csec) and collection latency (time taken to collect reward pellet in csec). All analyses were conducted using SPSS for Windows (version 19.0; SPSS, Chicago, IL). Dose-response data for each compound were analysed using separate repeated-measures analysis of variance (RM-ANOVA) with TREATMENT as a within-subject factor. A similar approach was also used for the infusion studies where each drug treatment was compared to its own vehicle control using a RM ANOVA with TREATMENT as a within-subject factor. Paired *t* tests were used to compare the effects of drug versus vehicle where only a single dose was tested (prelimbic infusions of the higher dose of LY341495 and each drug in the SSC experiments).

Graphs were plotted using Graphpad Prism 4.0 (Graphpad software, USA). Mauchly's test of sphericity was applied to repeated-measures analyses to correct the degrees of freedom to more conservative values using the Huynh-Feldt epsilon, for any instances of sphericity violation. Epsilon values (*ε*) are stated for any instance where the degrees of freedom have been corrected. Alpha level was set at equal to 0.05, with significant main effects being further analysed by post-hoc comparisons between vehicle and drug groups using least significant difference (LSD).

## Results

### Pre-treatment baseline

No significant difference was found for any variable across three consecutive baseline sessions prior to drug dosing indicating that the animals' performance was stable (Data not shown, F<1.63, *p*>0.219).

### Experiment 1: Effects of systemic treatment with NMDA antagonists on performance in a VITI 5CSRTT

**Figure 1:**
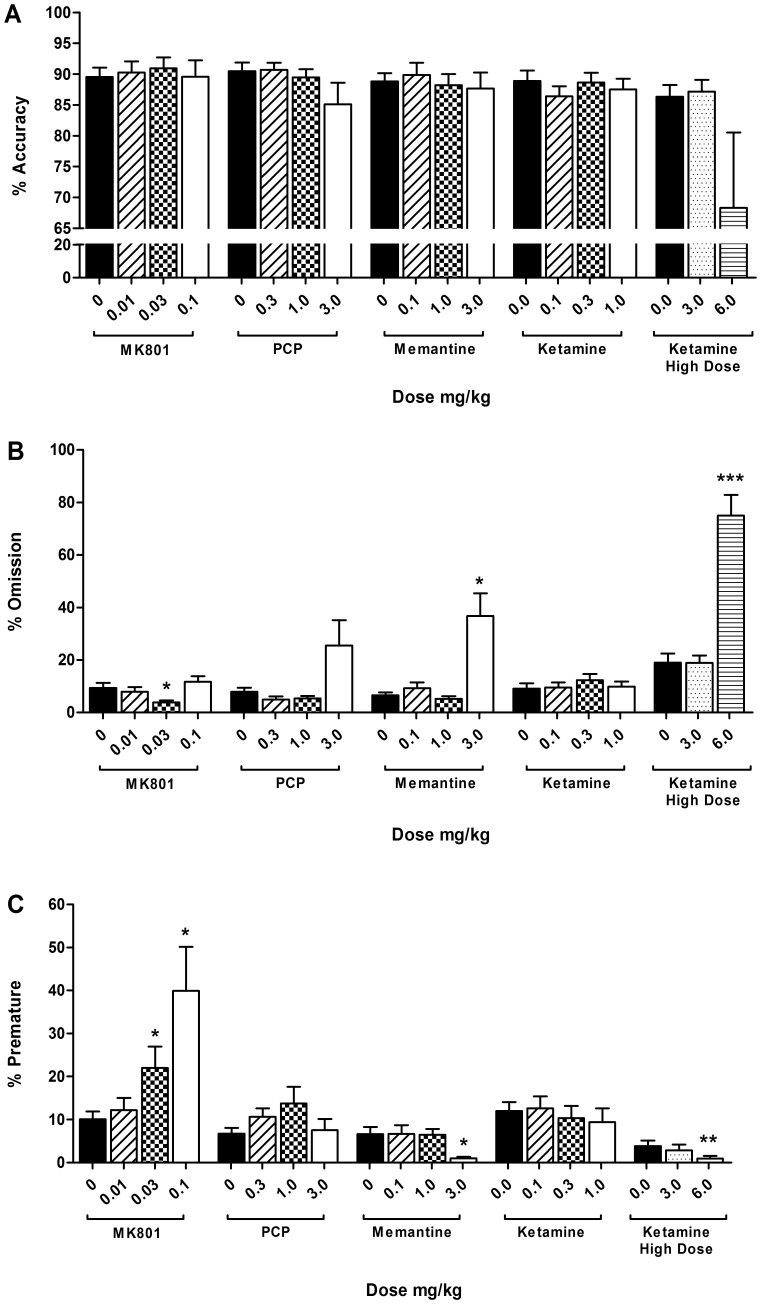
Effects of systemic NMDA antagonists in a VITI version of the 5CSRTT. The effects of MK801 (0.0–0.1 mg/kg, i.p.), PCP (0.0–3.0 mg/kg, i.p.), memantine (0.0–3.0 mg/kg, i.p.), and ketamine (0.0–6.0 mg/kg, i.p.) on accuracy (a), omissions (b), and premature responses (c) in a VITI version of the 5CSRTT. Results are shown for the total population, mean ± SEM, n = 12 animals per group, within subject, *p<0.05, **p<0.01, ***p<0.001 versus vehicle. 5CSRTT 5-choice serial reaction time task, VITI variable inter-trial interval.

Results for performance variables accuracy, omissions, and premature responses, are shown in Fig. 1. Correct responses and latency data are summarized in Table 1. Accuracy was not affected by NMDA antagonism in this VITI version of the 5CSRTT (Fig. 1a; MK801 F_[3.0, 33.0]_ = 0.22, *p* = 0.883; PCP F_[1.3, 13.8]_ = 1.67, *p* = 0.222, *ε* = 0.42; memantine F_[2.1, 22.6]_ = 0.59, *p* = 0.565, *ε* = 0.69; ketamine F_[3.0, 33.0]_ = 0.78, *p* = 0.513, n = 12).

**Table 1:** Effects of systemic NMDA antagonists on the number of correct responses and response latencies in a VITI version of the 5CSRTT.

Treatment	Dose (mg/kg)	Correct Responses	Correct latency (csec)	Collection latency (csec)
**MK801**	0.0	85.33±2.44	52.18±1.90	142.93±6.63
	0.01	87.42±2.79	50.37±1.96	**133.33±6.16** ***
	0.03	**91.92±2.32** ***	48.51±4.91	**129.55±7.09** ***
	0.1	79.67±5.60	54.17±12.04	**120.55±5.61** ***
**PCP**	0.0	87.58±2.26	52.83±1.61	148.03±6.94
	0.3	90.58±1.73	50.73±1.27	139.29±8.62
	1.0	88.92±1.55	53.59±1.72	134.39±6.59
	3.0	68.67±9.63	**64.74±3.34** *	166.61±18.56
**Memantine**	0.0	87.17±1.93	53.21±2.73	149.14±7.78
	0.1	85.75±3.02	53.96±2.33	144.99±7.20
	1.0	87.92±2.36	53.20±2.12	134.62±6.32
	3.0	**56.17±9.22** ***	**73.74±6.28** **	167.67±16.87
**Ketamine**	0.0	85.00±2.79	51.69±1.84	150.96±10.51
	0.1	81.50±3.13	50.68±1.47	145.77±10.09
	0.3	81.42±2.02	**54.76±1.88** *	149.02±8.94
	1.0	82.92±2.89	53.64±1.79	154.30±10.41
**High Dose**	0.0	73.58±3.98	59.93±3.31	156.71±10.35
	3.0	59.17±9.83	63.08±4.43	156.57±9.28
	6.0	**20.42±7.78** ***	56.93±10.61	137.44±26.24

The effect of MK801 (0.0–0.1 mg/kg, i.p.), PCP (0.0–3.0 mg/kg, i.p.), memantine (0.0–3.0 mg/kg, i.p.), and ketamine (0.0–6.0 mg/kg, i.p.) on correct responses and response latencies in a VITI version of the 5CSRTT. Results are shown for the total population, mean ± SEM, n = 12 animals per group, within-subject,

*p<0.05,

**p<0.01,

***p<0.001 versus vehicle.

5CSRTT 5-choice serial reaction time task, Csec centiseconds, VITI variable inter-trial interval.

MK801 treatment increased premature responses (Fig. 1c; F_[1.5, 16.0]_ = 5.02, *p* = 0.029, *ε* = 0.49, n = 12), with animals becoming more impulsive following 0.03 mg/kg (*p* = 0.034) and 0.1 mg/kg (*p* = 0.021) doses compared to vehicle treatment. MK801 also increased the number of correct responses (Table 1; F_[1.8, 19.3]_ = 4.47, *p* = 0.035, *ε* = 0.58, vehicle versus 0.03 mg/kg, *p* = 0.001, n = 12), and reduced the level of omissions (Fig. 1b; F_[3.0, 33.0]_ = 7.20, *p* = 0.001, vehicle versus 0.03 mg/kg, *p* = 0.006, n = 12). A dose dependent decrease in collection latency was also observed (Table 1; F_[2.2, 24.1]_ = 13.78, *p*<0.001, *ε* = 0.73, vehicle versus 0.01 mg/kg *p*<0.001, 0.03 mg/kg, p<0.001, 0.1 mg/kg, *p*<0.001, n = 12). Correct latency was not affected by MK801 treatment (F_[3.0, 33.0]_ = 2.50, *p* = 0.077, n = 12).

PCP treatment increased correct latency at the highest dose (Table 1; F_[1.7, 18.7]_ = 9.13, *p* = 0.002, vehicle versus 3.0 mg/kg, *p* = 0.013, *ε* = 0.57, n = 12). No other performance variables were affected (Fig. 1 & Table 1; correct responses F_[1.1, 11.8]_ = 3.94, *p* = 0.069, *ε* = 0.36; omissions F_[1.1, 11.6]_ = 4.12, *p* = 0.064, *ε* = 0.35; premature responses F_[2.1, 23.6]_ = 2.22, *p* = 0.128, *ε* = 0.71; collection latency F_[1.4, 15.5]_ = 3.49, *p* = 0.069, *ε* = 0.47).

Memantine treatment had no specific effects on task performance but caused an overall disruption to performance at the highest dose. This was evident by increased omissions (Fig. 1b; F_[1.1, 12.3]_ = 12.19, *p* = 0.004, *ε* = 0.37, vehicle versus 3.0 mg/kg, *p* = 0.004, n = 12), and reduced correct responses (Table 1; F_[1.1, 12.3]_ = 11.02, *p* = 0.005, *ε* = 0.37, vehicle versus 3.0 mg/kg, *p* = 0.006), and premature responses (Fig. 1c; F_[3.0, 33.0]_ = 5.15, *p* = 0.005, vehicle versus 3.0 mg/kg *p* = 0.006, n = 12). Correct latency was also increased (Table 1; F_[1.2, 13.2]_ = 11.68, *p* = 0.003, *ε* =  vehicle versus 3.0 mg/kg, *p* = 0.004, n = 12), whilst collection latency was unaffected (F_[1.5, 16.7]_ = 2.98, *p* = 0.089, *ε* = 0.40, n = 12).

Ketamine treatment increased correct latency (Table 1; F_[3.0, 33.0]_ = 3.25, *p* = 0.034, 0.3 mg/kg versus vehicle, *p* = 0.024, n = 12) but had no other effects on other performance variables (Fig. 1 & Table 1; correct responses F_[3.0, 33.0]_ = 1.00, *p* = 0.408; omissions F_[3.0, 33.0]_ = 1.62, *p* = 0.203; premature F_[1.7, 19.0]_ = 0.48, *p* = 0.600, *ε* = 0.58; collection latency F_[3.0, 33.0]_ = 0.54, *p* = 0.656). The higher dose of ketamine increased omissions (Fig. 1b; F_[1.3, 14.6]_ = 13.82, *p* = 0.001, *ε* = 0.66, vehicle versus 6.0 mg/kg, *p*<0.0001, n = 12), and reduced the number of correct responses (Table 1; F_[1.3, 14.8]_ = 11.13, *p* = 0.003, *ε* = 0.67, vehicle versus 6.0 mg/kg *p*<0.0001, n = 12), and premature responses (Fig. 1c; F_[3.0, 33.0]_ = 9.29, *p*<0.0001, vehicle versus 6.0 mg/kg, *p* = 0.004, n = 12). Response latencies were not affected (Table 1; correct latency F_[1.2, 12.8]_ = 0.21, *p* = 0.219, *ε* = 0.58; collection latency F_[1.3, 14.7]_ = 0.43, *p* = 0.577, *ε* = 0.67, n = 12).

### Experiment 2: Effects of systemic treatment with NMDA antagonists during a short stimulus challenge in the VITI 5CSRTT

**Figure 2:**
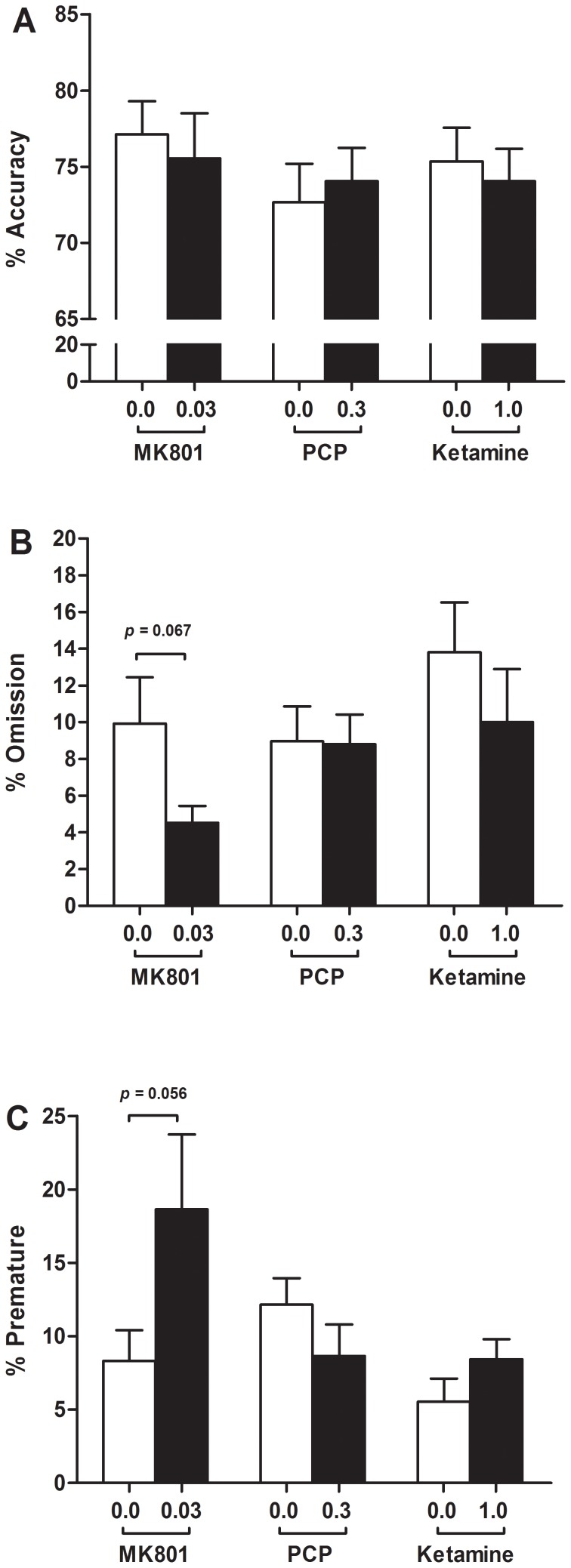
Effects of systemic NMDA antagonists during a short stimulus challenge. The effect of MK801 (0.03 mg/kg, i.p.), PCP (0.3 mg/kg, i.p.), and ketamine (1.0 mg/kg, i.p.) on accuracy (a), omissions (b), and premature responses (c), in animals trained in a VITI version of the 5CSRTT during an acute attentional challenge. Results are shown for the total population, mean ± SEM, n = 12 animals per group, within subject. 5CSRTT 5-choice serial reaction time task, VITI variable inter-trial interval.

Fig. 2 shows accuracy, omissions, and premature responses, for single doses of ketamine (1.0 mg/kg), MK801 (0.03 mg/kg), and PCP (0.3 mg/kg) versus vehicle treated controls. The number of correct responses and latency data are summarized in Table 2. Animals showed an approximate 15% reduction in accuracy compared to baseline performance (0.25 s versus 0.5 s stimulus duration). No other parameters were affected by the change in stimulus duration (data not shown).

**Table 2:** Effects of systemic NMDA antagonists on the number of correct responses and response latencies during a short stimulus challenge.

Treatment	Dose (mg/kg)	Correct Responses	Correct latency (csec)	Collection latency (csec)
**MK801**	0.0	73.17±3.39	55.73±3.01	145.84±10.35
	0.03	75.83±3.31	**48.30±2.31** *	**128.31±8.25** **
**PCP**	0.0	67.25±2.92	57.64±4.11	146.16±4.57
	0.3	68.75±3.02	54.64±2.22	137.84±5.90
**Ketamine**	0.0	68.25±3.04	54.09±2.21	144.20±6.25
	1.0	69.67±4.00	54.42±2.57	136.43±6.63

The effect of MK801 (0.03 mg/kg, i.p.), PCP (0.3 mg/kg, i.p.), and ketamine (1.0 mg/kg, i.p.) on correct responses and response latencies in a VITI version of the 5CSRTT under an acute attentional challenge. Results are for the total population, mean ± SEM, n = 12 animals per group, within-subject,

*p<0.05,

**p<0.01.

5CSRTT 5-choice serial reaction time task, Csec centiseconds, VITI variable inter-trial interval.

Accuracy was not affected by any of the drugs tested (Fig. 2a; MK801 *t*_[11]_ = 0.6, *p* = 0.586; PCP *t*_[11]_ = −0.8, *p* = 0.450; ketamine *t*_[11]_ = 0.7, *p* = 0.485, n = 12). MK801 reduced correct latency (Table 2; *t*_[11]_ = 2.9, *p* = 0.014, n = 12) and collection latency (*t*_[11]_ = 3.9, *p* = 0.002, n = 12), but had no effects on omissions (Fig. 2b; *t*_[11]_ = 2.0, *p* = 0.067, n = 12), premature responses (Fig. 2c; *t*_[11]_ = −2.1, *p* = 0.056, n = 12), or correct responses (*t*_[11]_ = −0.90, *p* = 0.389, n = 12).

PCP and ketamine had no effects on any behavioural parameters (Fig. 2 & Table 2; PCP: correct responses *t*_[11]_ = −0.49, *p* = 0.636; omissions *t*_[11]_ = −0.3, *p* = 0.750; prematures *t*_[11]_ = −1.2, *p* = 0.260; correct latency *t*_[11]_ = 0.9, *p* = 0.411; collection latency *t*_[11]_ = 1.7, *p* = 0.112, n = 12; ketamine: correct responses *t*_[11]_ = −0.35, *p* = 0.731; omissions *t*_[11]_ = 1.1, *p* = 0.295; prematures *t*_[11]_ = −1.6, *p* = 0.136; correct latency *t*_[11]_ = −0.2, *p* = 0.880; collection latency, *t*_[11]_ = 1.7, *p* = 0.112, n = 12).

### Experiment 3: Effects of glutamate receptor blockade in prelimbic versus infralimbic cortex in a VITI 5CSRTT

**Figure 3:**
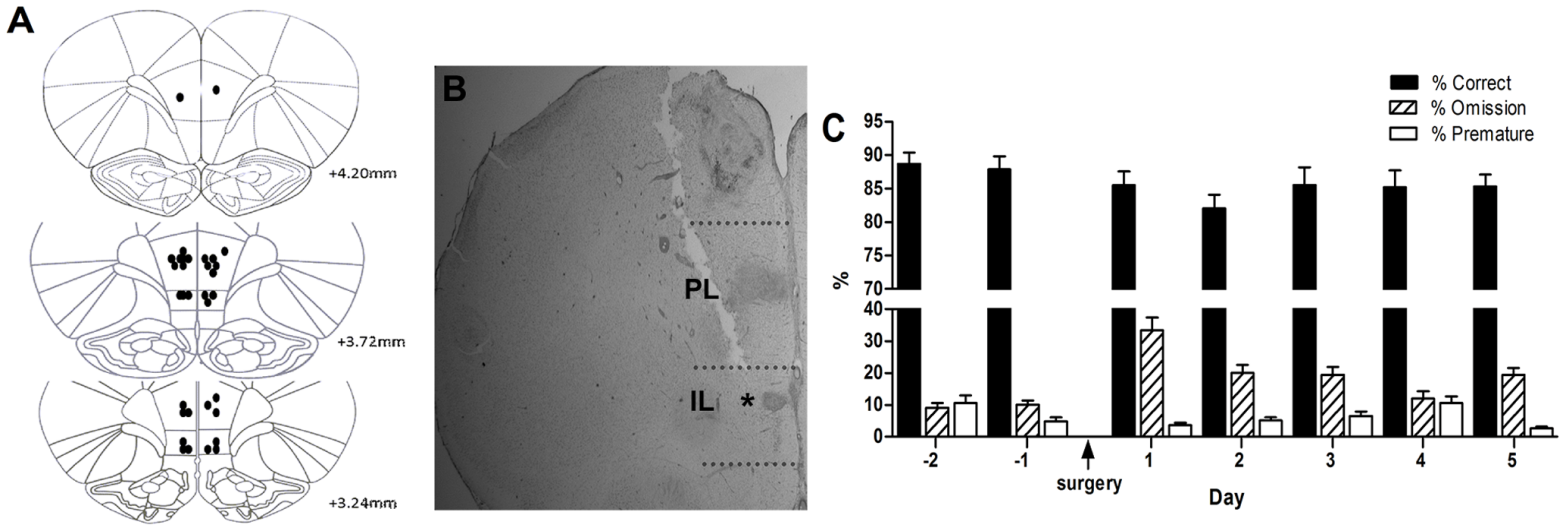
Injector tip placement for mPFC infusions and pre- and post-operative baseline performance. Schematic diagrams showing the location of the injector tips (a) in the prelimbic (n = 10) and infralimbic cortices (n = 6), reconstructed from Paxinos and Watson (2007). Representative image of the mPFC showing the position of the infralimbic injection site (b, asterisk). Pre- and post-operative baseline performance (c), data presented for the total population, n = 12, mean ± SEM. mPFC medial prefrontal cortex, PL prelimbic cortex, IL infralimbic cortex.

Fig. 3b shows the final injector tip placement in the infralimbic cortex. Due to the subsequent tract damage caused by the infralimbic injectors, the position of the prelimbic injectors was estimated from this final location (Fig. 3a). Animal numbers for the final analysis were as follows; prelimbic, MMPIP n = 10, LY341495 and MK801 n = 9, infralimbic n = 6 for all drugs. After surgery, animals returned to criterion (>80% accuracy, <20% omissions) within the 5 days of postoperative re-baseline sessions (Fig. 3c).

**Figure 4:**
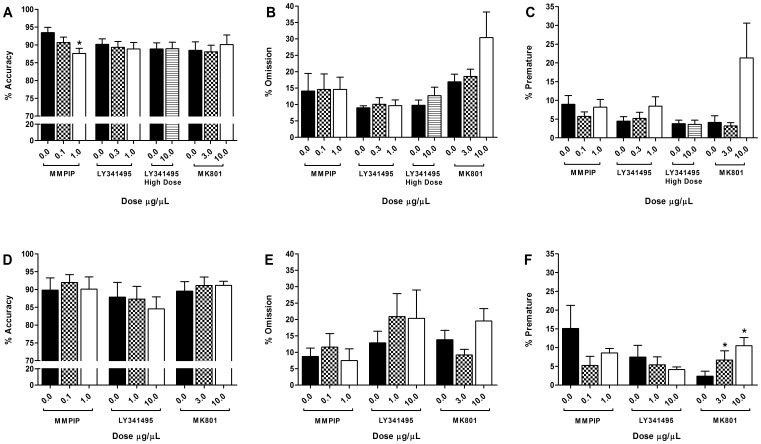
Effects of MMPIP, LY341495, and MK801 infusions in a VITI version of the 5CSRTT. The effect of intracerebral infusions into the prelimbic (a–c) and infralimbic (d–f) cortices for MMPIP (0.0–1.0 µg/µL), LY341495 (0.0–10.0 µg/µL), and MK801 (0.0–10.0 µg/µL) on accuracy (a, d), omissions (b, e) and premature responses (c, f) in a VITI version of the 5CSRTT. Results are shown for the total population, mean ± SEM, animals per group prelimbic: n = 10 (MMPIP), n = 9 (LY341495, MK801), infralimbic n = 6, within subject, *p<0.05 versus vehicle. 5CSRTT 5-choice serial reaction time task, VITI variable inter-trial interval.

Fig. 4 shows accuracy, omissions, and premature response data following infusions of MMPIP, LY341495, and MK801, within the prelimbic (a–c) and infralimbic (d–f) cortices. The number of correct responses as well as latency data are summarized in Table 3.

**Table 3:** Effects of MMPIP, LY341495, and MK801, mPFC infusions on the number of correct responses and response latencies in a VITI version of the 5CSRTT.

Treatment	Dose (µg/µL)	Correct Responses	Correct latency (csec)	Collection latency (csec)
**Prelimbic Cortex**				
**MMPIP**	0.0	84.00±5.20	58.81±3.71	149.81±5.70
	0.1	81.30±5.10	60.41±5.22	155.65±6.13
	1.0	78.60±3.95	60.89±3.72	152.95±9.37
**LY341495**	0.0	86.11±1.15	58.23±2.45	157.89±8.01
	0.3	84.33±2.39	57.54±3.86	160.13±6.63
	1.0	84.33±2.48	53.19±2.16	152.78±6.71
**High Dose**	0.0	81.11±4.06	62.39±3.43	158.13±5.46
	10.0	82.67±3.33	55.32±1.82*	156.57±7.00
**MK801**	0.0	77.11±2.90	57.33±2.40	159.09±8.23
	3.0	75.44±3.01	61.35±2.61	162.29±9.86
	10.0	58.44±10.41	69.10±8.23	150.46±4.88
**Infralimbic Cortex**				
**MMPIP**	0.0	86.17±4.50	54.53±4.14	149.89±10.78
	0.1	85.50±5.07	54.92±4.43	161.81±7.04*
	1.0	88.17±6.31	52.27±4.83	150.32±8.60
**LY341495**	0.0	80.17±5.04	58.76±5.00	156.90±7.07
	1.0	72.67±8.02	60.48±5.92	169.70±10.97
	10.0	71.67±9.30	59.56±4.73	167.86±10.10
**MK801**	0.0	81.33±4.85	59.82±4.24	169.82±13.54
	3.0	87.00±3.59	55.47±3.58	149.15±9.80
	10.0	77.00±3.66	56.41±2.32	143.54±5.63

The effect of intracerebral infusions into the prelimbic and infralimbic cortices of MMPIP (0.0–1.0 µg/µL), LY341495 (0.0–10.0 µg/µL), and MK801 (0.0–10.0 µg/µL) on correct responses and response latencies in a VITI version of the 5CSRTT. Results are shown for the total population, mean ± SEM, prelimbic MMPIP n = 10, LY341495 and MK801 n = 9, infralimbic n = 6 animals per group, within-subject, *p<0.05 versus vehicle.

5CSRTT 5-choice serial reaction time task, Csec centiseconds, VITI variable inter-trial interval.

MK801 infusions into the prelimbic or infralimbic cortices had no effect on accuracy (Fig. 4a, d; prelimbic F_[1.3, 10.4]_ = 0.27, *p* = 0.679, *ε* = 0.65, n = 9; infralimbic F_[2.0, 10.0]_ = 0.47, *p* = 0.559, n = 6). A dose dependent increase in premature responses was found following infralimbic (Fig. 4f; F_[2.0, 10.0]_ = 11.75, *p* = 0.002, 3 µg/µL versus vehicle *p* = 0.038, 10 µg/µL versus vehicle *p* = 0.002, n = 6), but not prelimbic infusions (Fig. 4c; F_[1.1, 8.6]_ = 3.45, *p* = 0.096, *ε* = 0.54, n = 9). No other effects on performance were found for prelimbic (Fig. 4 & Table 3; omissions F_[1.1, 9.1]_ = 2.57, *p* = 0.142, *ε* = 0.57; correct responses F_[1.0, 8.2]_ = 2.80, *p* = 0.131, *ε* = 0.52; correct latency F_[1.3, 10.1]_ = 1.85, *p* = 0.207, *ε* = 0.63; collection latency F_[2.0, 16.0]_ = 2.23, *p* = 0.140, n = 9), or infralimbic infusions (Fig. 4 & Table 3; omissions F_[1.2, 5.8]_ = 3.14, *p* = 0.127, *ε* = 0.58; correct responses F_[2.0, 10.0]_ = 2.69, *p* = 0.116; correct latency F_[2.0, 10.0]_ = 1.00, *p* = 0.403; or collection latency F_[2.0, 10.0]_ = 2.50, *p* = 0.132, n = 6).

Blockade of mGluR7 using MMPIP appeared to specifically impair accuracy when infused into the prelimbic but not infralimbic cortex (Fig. 4a, d; prelimbic F_[2.0, 18.0]_ = 4.75, *p* = 0.022, 1.0 µg/µL versus vehicle *p* = 0.010, n = 10; infralimbic F_[2.0, 10.0]_ = 1.33, *p* = 0.308, n = 6). Accuracy affects were found in the absence of effects on other performance variables for the prelimbic cortex (correct responses F_[2.0, 18.0]_ = 1.18, *p* = 0.331; omissions F_[1.3, 11.5]_ = 0.02, *p* = 0.940, *ε* = 0.64; premature responses F_[1.4, 12.4]_ = 1.02, *p* = 0.361, *ε* = 0.69; correct latency F_[1.4, 12.2]_ = 0.21, *p* = 0.725, *ε* = 0.68; collection latency F_[2.0, 18.0]_ = 0.77, *p* = 0.480, n = 10). Following infralimbic infusions, MMPIP increased collection latency at a dosage of 0.1 µg/µL but had no effect at the higher dose of 1.0 µg/µL (Table 3; F_[2.0, 10.0]_ = 8.45, *p* = 0.007, 0.1 µg/µL versus vehicle *p* = 0.028, n = 6). No other effects were observed (omissions F_[2.0, 10.0]_ = 1.33, *p* = 0.308; prematures F_[2.0, 10.0]_ = 2.15, *p* = 0.167; correct responses F_[2.0, 10.0]_ = 0.25, *p* = 0.787; correct latency F_[2.0, 10.0]_ = 0.62, *p* = 0.557, n = 6).

Infusion of the mGluR2/3 antagonist, LY341495 into the prelimbic cortex had no effect on any performance measures (Fig. 4 & Table 3; accuracy F_[2.0, 16.0]_ = 0.32, *p* = 0.730; premature responses F_[1.3, 10.2]_ = 2.41, *p* = 0.149, *ε* = 0.64; omissions F_[2.0, 16.0]_ = 0.19, *p* = 0.831; correct responses F_[2.0, 16.0]_ = 0.33, *p* = 0.727; correct latency F_[2.0, 16.0]_ = 3.09, *p* = 0.074; collection latency F_[2.0, 16.0]_ = 0.58, *p* = 0.571; n = 9). Infusing a higher dose of LY341495 decreased correct latency (*t*_[8]_ = 2.63, *p* = 0.030), with no further effects on any other variables (accuracy *t*_[8]_ = 0.27, *p* = 0.792; correct responses *t*_[8]_ = −0.37, *p* = 0.721; omissions *t*_[8]_ = 0.28, *p* = 0.789; prematures *t*_[8]_ = 0.86, *p* = 0.416; collection latency *t*_[8]_ = 0.24, *p* = 0.816, n = 9). Infralimbic infusions of LY341495 showed no effect on performance (Fig. 4 & Table 3; accuracy F_[2.0, 10.0]_ = 0.65, *p* = 0.541; correct responses F_[2.0, 10.0]_ = 1.05, *p* = 0.384; omissions F_[2.0, 10.0]_ = 1.19, *p* = 0.343; premature responses F_[2.0, 10.0]_ = 0.63, *p* = 0.551; correct latency F_[2.0, 10.0]_ = 0.08, *p* = 0.923; collection latency F_[2.0, 10.0]_ = 1.91, *p* = 0.198, n = 6).

## Discussion

The VITI 5CSRTT used in these studies had previously been shown to have a greater sensitivity to the cognitive enhancing effects of atomoxetine and methylphenidate [32]. We therefore hypothesised that this task may also provide a more sensitive method to study the cognitive impairments seen following acute administration of NMDA antagonists [9], [13], [15]. Contrary to our hypothesis, the NMDA antagonists tested here did not induce any specific impairments in attention and only MK801 increased impulsive responding. When we further increased the attentional demands of the task by reducing the stimulus duration, we also failed to see any deficits following acute blockade of NMDA receptors. At higher doses, the NMDA antagonists all impaired general performance in the task. This was reflected by increases in omitted trials and increased latencies to respond and collect reward, an effect which may relate to the sedative or locomotor disrupting effects of these drugs at higher doses [10], [34], [35]. In the infusion studies, infralimbic blockade of NMDA receptors with MK801 resulted in an increase in premature responding which was not seen during prelimbic infusions. This effect is similar to that reported by Murphy et al following r-CPP infusions, and further supports the hypothesis that the infralimbic but not prelimbic cortex is a key region involved in regulating impulsivity [13], [14]. However, in contrast to previous studies we did not find any impairment in visuo-spatial attention following prelimbic blockade of NMDA receptors using MK801 [18], [20], [36]–[38]. LY341495 also failed to have any effects although MMPIP appeared to induce an impairment in accuracy following prelimbic but not infralimbic infusions although this result requires further investigation. The following discussion considers the findings from these different experiments and glutamate-mediated regulation of attention and impulse control in the 5CSRTT.

### Effects of acute blockade of NMDA receptors and performance in a VITI version of the 5CSRTT

Previous studies have proposed that increased cortical glutamate release is associated with NMDA-mediated deficits in attention and impulse control in the 5CSRTT [18]. Interestingly, none of the drugs tested here showed any specific effect on visuo-spatial attention despite being used over a range of doses comparable with previous studies [4], [15], [39]. At higher doses, animals exhibited a general impairment in performance but no specific attentional deficits were seen. This included the study where the attentional demands of the task were increased using a short stimulus challenge. Across the literature, the use of acute treatment with NMDA antagonists to induce a cognitive impairment in animal performing the 5CSRTT has yielded conflicting data [4]. In the majority of studies, animals are shown to exhibit reduced attentional accuracy but the same dose also impairs omissions and response latencies suggesting a more general disruption of performance [4], [15], [16], [40], [41]. This was something we had hoped to address by introducing a more challenging task format. Contrary to our prediction, animals performing this modified version of the 5CSRTT, where, stimuli were presented in a less predictable manner, appeared less impaired. Although we have not made a direct comparison between groups of animals trained in the two tasks, we suggest that a possible reason for the reduced effects of NMDA receptor blockade may result from differences in the way the rats perform our task. Previous studies have suggested that rodents trained in tasks, such as the 5CSRTT with a fixed ITI, develop behavioural strategies to facilitate performance [2], [12], [26]. These strategies usually involve some sequence of behaviours or motor patterns which may provide a means for animals to time their behaviour. If this is the case in the fixed ITI 5CSRTT, then any drug treatment which disrupts these behaviours would also lead to an impairment in performance. In the VITI version of the task, the ability of the animals to use such behavioural strategies is reduced by the more unpredictable presentation of the stimulus, potentially increasing the attentional demands of the task. We proposed that this contributed to the enhanced sensitivity we and others have observed when using VITI tasks and cognitive enhancers [27], [32]. It may also mean that the disrupting effect of NMDA receptor antagonism involves, at least in part, some effects on these behavioural strategies. By reducing the development of these behaviours using a VITI task we may also have reduced the attentional impairments induced by NMDA antagonists. The effects of systemically administered NMDA antagonists on behavioural strategies may also reflect actions in brain regions other than the mPFC, hence why prelimbic infusions of MK801 also lacked effects [42], [43].

It was also interesting to observe that our NMDA antagonists did not all induce similar behavioural effects in this task. This was despite our attempts to test them at doses, and over a time course which reflected comparable blockade of the receptor. MK801 appeared to have a stimulant-like effect with reductions in latencies, increased premature responses and increased correct trials. In contrast, ketamine and memantine and to some extent, PCP, reduced the animal's motivation to perform the task with higher doses increasing latencies, and for ketamine and memantine, increasing omissions. All the drugs tested were non-competitive NMDA receptor antagonists which are used relatively interchangeably in the literature to impair cognition in animals [9], [10], [15], [44]. However, the individual pharmacological profiles of the drugs do differ which may account for these difference [45]. It is also possible that the affinity for the receptor and time course of administration were not fully matched in this study. The results presented here suggest that NMDA antagonism may have a greater effect in the fixed ITI 5CSRTT because of the way the animal performs the task as opposed to inducing a specific attentional impairment. Studies in other cognition models have also found inconsistent effects with NMDA antagonists [10], suggesting these studies may not fully replicate the type of cognitive impairments induced by NMDA antagonism in humans [1], [46], although doses were adjusted to try to take these factors into account. Taken together, these findings suggests acute systemic NMDA antagonists as a model of hypo-glutamatergic function in animals may not provide reliable cognitive impairments, and protocols using sub-chronic dosing regimens may be more relevant to the effects observed in humans [3], [9], [41].

### Effects of prelimbic versus infralimbic targeting of glutamate receptors and performance in a VITI version of the 5CSRTT

Previous studies have shown that the mPFC plays an important role in modulating behaviour in the 5CSRTT [47]–[49]. Direct infusion of the NMDA antagonist, r-CPP induces dissociable effects on attention and impulse control involving the prelimbic and infralimbic regions respectively [13]. Subsequent studies have attempted to resolve the mechanisms which underlie these effects including studies where glutamate transmission has been disrupted using re-uptake inhibitors and AMPA antagonists [14]. Similar to previous studies, we observed that infusion of the NMDA antagonist, MK801 in the infralimbic but not prelimbic cortex, was associated with an impairment in impulse control. However, we did not find any deficits in attention following prelimbic infusion. It may be that, in a similar manner to the effects seen with systemic administration of NMDA antagonists, changing the format of the task also impacts on sensitivity to prelimbic manipulations which disrupt NMDA function [2], [12], [26].

Based on our data, the effects on performance mediated by NMDA receptor antagonism may be different from those where the regulation of glutamate release is altered through targeting pre-synaptic metabotropic glutamate receptors. In our study, reducing the function of mGluR7 using the negative allosteric modulator, MMPIP, resulted in an impairment in visuo-spatial attention which was specific to the prelimbic cortex. The mGluR2/3 antagonist had no effect when infused into either region and MMPIP lacked any effects following infralimbic infusion. Although the possibility exists that the lack of effect arose from repeated infusions into the same region leading to damage and the loss of the relevant structures, our results do not support this. In our final infusion experiment, MK801 reduced premature responding consistent with previous findings (Murphy et al., 2005). This study was relatively limited in terms of the number of animals used and number of infusions carried out and as such only tentative conclusions can been drawn. In our experiment design, we also treated each dose-response study as an independent experiment, carrying out separate RM ANOVA analyses, which increases the risk of a type I error. However, our results suggest that glutamate modulation through mGluR7 but not mGluR2/3 or NMDA receptors, has a greater effect on visuo-spatial attention in the VITI task. This suggests that the way in which glutamate transmission is modulated may differentially affect attentional processes, which is also task dependent. In particular, the importance of PFC-striatal interactions for visuo-spatial attention under a fixed ITI has previously been demonstrated [18], [42].

In summary, these studies suggest that NMDA antagonists do not induce a specific cognitive impairment in our VITI 5CSRTT. It is possible that the way the animals learn to perform the task and the degree to which a behavioural strategy is adopted may impact on the effects of NMDA antagonism. This may to some extent explain the high degree of variability seen in the literature. It would be interesting to also test an alternative variant of the 5CSRTT, the 5C-CPT [24], [50], which has recently been developed and tested in both rodents and humans [51]–[54]. This task more closely reflects the human continuous performance task and is sensitive to NMDA antagonism [55], cognitive enhancers [54], and drugs targeting the cholinergic system [53], [56]. Further studies are also needed to build on the infusion data presented here however, the results with MMPIP versus MK801, may relate to the way in which glutamate regulation is altered by drug treatment. This could raise the possibility that drugs which facilitate mGluR7 could have beneficial effects in attention.
